# Synthetic Imine Resveratrol Analog 2-Methoxyl-3,6-Dihydroxyl-IRA Ameliorates Colitis by Activating Protective Nrf2 Pathway and Inhibiting NLRP3 Expression

**DOI:** 10.1155/2019/7180284

**Published:** 2019-11-21

**Authors:** Yeru Chen, Zhaohong Zheng, Chang Li, Yuanjiang Pan, Xiuwen Tang, Xiu Jun Wang

**Affiliations:** ^1^Department of Pharmacology and Cancer Institute of the Second Affiliated Hospital, Zhejiang University School of Medicine, Hangzhou 310058, China; ^2^Department of Chemistry, Zhejiang University, 38 Zheda Road, Hangzhou 310027, China; ^3^Department of Biochemistry and Department of Thoracic Surgery of the First Affiliated Hospital, Zhejiang University School of Medicine, Hangzhou 310058, China

## Abstract

Resveratrol (RSV) is a naturally occurring polyphenol that exhibits pleiotropic health benefits, including anticolitis and colon cancer-protective activity. Recently, we identified the novel imine RSV analog (IRA), 2-methoxyl-3,6-dihydroxyl-IRA 3,4,5,4-tetramethoxystilbene (C33), as a putative activator of nuclear factor erythroid 2-related factor 2 (Nrf2). The present study was designed to evaluate the ability of C33 to activate the Nrf2 signaling pathway and its anticolitis effect in comparison to RSV. The anticolitis action of C33 was assessed in a mouse model of colitis induced by dextran sulfate sodium (DSS). The effect of C33 on the Nrf2 signaling pathway was examined *in vitro* and *in vivo*. Compared to RSV, C33 triggered a more dramatic increase in the expression of genes downstream of Nrf2 in LS174T cells as well as in the small intestine and colon of wild-type (WT) mice. Correlated with its superior ability to activate the cytoprotective Nrf2 pathway, C33 was significantly better in ameliorating DSS-induced colitis by improving the inflammation score, as well as downregulating the markers of inflammation in WT mice. Moreover, induction of the NOD-like receptors family pyrin domain containing 3 (NLRP3) inflammasome by colitis was also significantly inhibited by the IRA. Nrf2 knockout completely abolished the effects of C33, indicating that Nrf2 is the important mechanistic target of C33 *in vivo*. In conclusion, the novel IRA, C33, has stronger anticolitis effects than RSV. Further studies are warranted to evaluate C33 as a potential therapeutic agent for inflammatory bowel disease and cancer chemoprevention.

## 1. Introduction

Colorectal cancer is a life-threatening disease that can develop spontaneously or as a complication of inflammatory bowel disease. Chronic inflammation, as in ulcerative colitis and Crohn's disease, has been strongly linked to an increased risk of colorectal cancer [1]. Conventional treatments of colitis can reduce the periods of active disease and help to maintain remission, but such treatments often have marginal results, patients become refractory, and there are side-effects. There is a need for the development of alternative treatment strategies for inflammatory bowel disease.

Nuclear factor erythroid 2-related factor 2 (Nrf2) is a master regulator of cellular defenses against oxidative and electrophilic stress, toxic chemicals, and carcinogens [2–4]. Under basal homeostatic conditions, Nrf2 is degraded rapidly by the ubiquitin-proteasome pathway *via* its association with Keap1 (Kelch-like ECH-associated protein 1). Upon exposure to oxidative or electrophilic stress, reactive cysteine residues in Keap1 are covalently modified, liberating Nrf2 from Keap1-mediated degradation [5, 6]. This results in translocation of Nrf2 to the nucleus where it binds with antioxidant response elements (AREs) in the promoter region of its target genes, thereby inducing a battery of cytoprotective genes and antioxidative enzymes [7]. The ARE-driven genes include glutathione S-transferases (GSTs), NAD(P)H:quinone oxidoreductase (NQO1), aldo-keto reductase (AKR), and heme oxygenase-1 (HO-1) [8, 9]. Constitutive Nrf2 signaling activity has shown to be positively correlated with maximum lifespan potential and that this activity was also manifested in high levels of downstream gene expression and activity in naturally long-lived rodents [10]. Nrf2 also plays a key anti-inflammatory role by downregulating cytokines, chemokines, cell adhesion molecules, matrix metalloproteinases, and inducible nitric oxide synthase, as well as inhibiting the activation of NOD-like receptors family pyrin domain containing 3 (NLRP3) inflammasome [11]. Many natural compounds with chemopreventive properties have been identified as Nrf2 activators [12]. Natural compounds, such as epigallocatechin gallate, sulforaphane, resveratrol (RSV), lycopene, and green tea extract, have therapeutic effects on inflammatory diseases by activating the Nrf2 signaling pathway [13]. Nrf2-deficient mice have an increased susceptibility to dextran sulfate sodium- (DSS-) induced colitis and colitis-associated colorectal cancer [14–16]. Using animal models, Nrf2 activators have been shown to be promising for abating colitis and preventing colitis-associated colon cancer [17–20].

RSV, a dietary antioxidant polyphenol found in grapes, red wine, and peanuts, has protective effects against cardiovascular disease and, particularly, against inflammation and all stages of carcinogenesis [21, 22]. The polyphenol has beneficial effects as antiaging compounds through modulating the hallmarks of aging, including oxidative damage, inflammation, telomere attrition, and cell senescence [23–29]. Animal studies have demonstrated that RSV has anti-inflammatory effects on several organ systems [30, 31], including the gastrointestinal tract [32, 33]. In the azoxymethane/DSS mouse model of colitis-associated colon cancer, RSV supplementation decreased the tumor incidence from 80% to 20%, as well as the tumor number. RSV is regarded as a useful, nontoxic, complementary, and alternative strategy to abate colitis and potentially colon cancer associated with colitis [32].

It is widely recognized that RSV is a multitargeting agent [34]. An important mechanistic feature is that RSV is thought to contribute to its cancer chemopreventive activity through its ability to activate the protective Nrf2/ARE pathway [35, 36]. With the aim of optimizing the potency of RSV in activating the Nrf2/ARE pathway, in previous studies we synthesized an array of synthetic polyphenols, imine RSV analogs (IRAs) [37]. Among the IRAs, we identified 2-methoxyl-3,6-dihydroxyl-IRA, defined as C33, as the most potent putative Nrf2 activator [38]. Here, we carried out further investigations to evaluate the ability of this compound to activate the endogenous Nrf2/ARE signaling pathway *in vitro* and *in vivo* and to compare the anticolitis properties of C33 with those of the naturally occurring molecule.

## 2. Materials and Methods

### 2.1. Chemicals and Reagents

**Figure 1:**
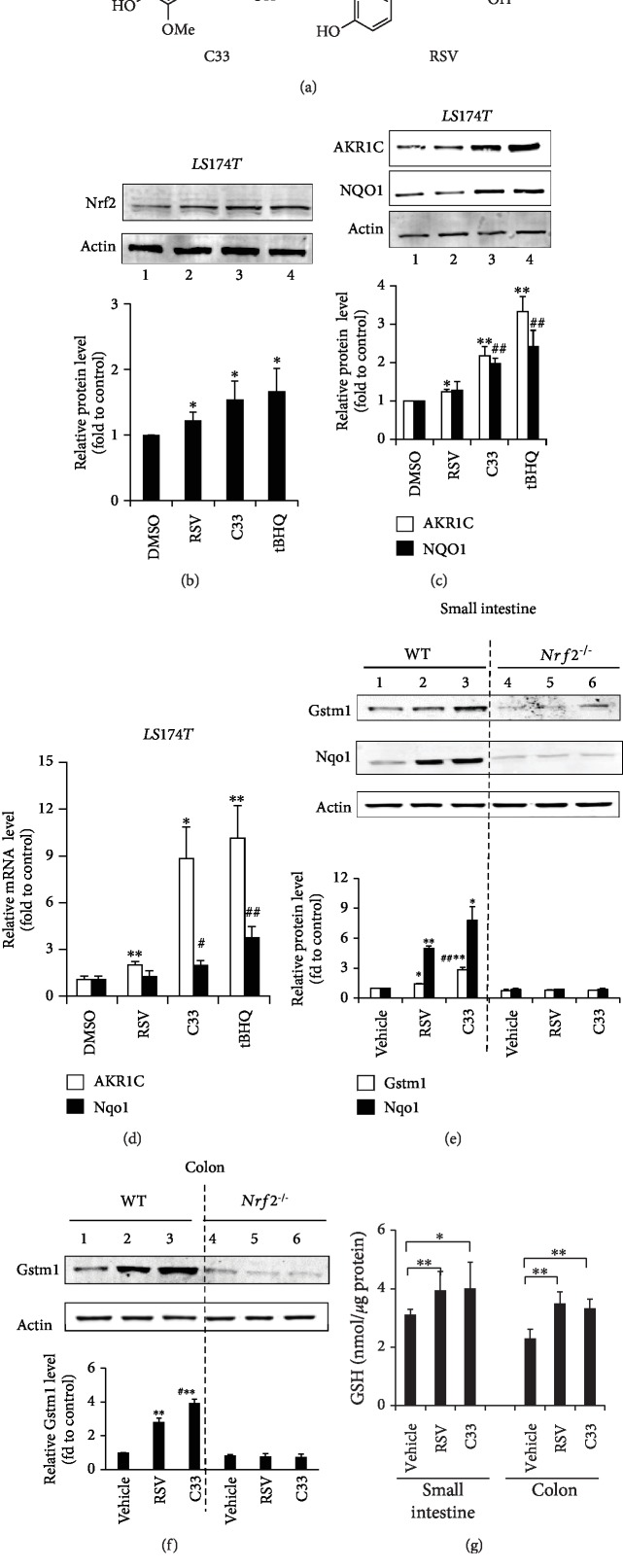
C33 is more potent in activating Nrf2 target genes than RSV *in vitro*. (a) The structures of C33 and RSV. (b–d) C33 increased the expression of Nrf2, AKR1C, and NQO1 in LS174T cells more strongly than RSV. LS174T cells were treated with C33 (1 *μ*M), RSV (5 *μ*M), or tBHQ (20 *μ*M) for 24 h. The cell lysates were analyzed by Western immunoblotting with antibodies against Nrf2 (b), AKR1C, or NQO1 (c). Actin was used as a loading control and tBHQ treatment served as a positive control. (b, c) Upper panel, representative images of Western immunoblots. Lower panel, semiquantitative result of blot. The value from cells treated with DMSO (control) was set at 1. Values are mean ± SD (*n* = 3). ^∗^*p* < 0.05, ^∗∗^*p* < 0.01, compared with cells treated with DMSO with same protein. ^#^*p* < 0.05, ^##^*p* < 0.01, compared with cells treated with RSV with same protein. (d) The mRNA levels of AKR1C1 and NQO1were determined by Taqman RT-PCR analysis. The level of 18S rRNA was used as internal control. The value from cells treated with DMSO (control) was set at 1. Values are mean ± SEM (*n* = 3). ^∗^*p* < 0.05, ^∗∗^*p* < 0.01, compared with cells treated with DMSO with same mRNA. ^#^*p* < 0.05, ^##^*p* < 0.01, compared with cells treated with RSV with same mRNA. (e–g) C33 is more potent than RSV in increasing the expression of ARE-driven genes in the small intestine and colon from WT but not *Nrf2^−/−^* mice. WT and *Nrf2^−/−^* mice were given C33 (200 mg/kg i.g.), RSV (200 mg/kg i.g.), or 10% ethanol (vehicle) for 3 days. Soluble extracts from the small intestine (e) and (f) colon were analyzed by Western immunoblotting with antibodies against Gstm1 or Nqo1. (e, f) Upper panel, representative images of Western immunoblots. Each lane shows the results for a sample from a single mouse. Actin was used as a loading control. Lower panel, semiquantitative result of blot. The value for the same protein from WT mice treated with vehicle (control) was set at 1. Values are mean ± SD (*n* = 3). ^∗^*p* < 0.05, ^∗∗^*p* < 0.01, compared with vehicle-treated mice with same protein and phenotype. ^#^*p* < 0.05, ^##^*p* < 0.01, compared with RSV-treated mice with same protein and phenotype. (g) Glutathione (GSH) levels in the colon and small intestine from WT mice treated with RSV or C33. Values are mean ± SD (*n* = 6–9); ^∗^*p* < 0.05, ^∗∗^*p* < 0.01, compared with vehicle-treated WT mice (control). Blots in (b), (c), (e), and (f) represent results from at least three independent experiments.

Unless otherwise stated, all chemicals were from Sigma-Aldrich Co., Ltd. (Shanghai, China). Antibodies against nitrotyrosine and Nrf2 (H300; sc-13032) were from Santa Cruz Biotechnology (Shanghai, China). Gstm1 (glutathione S-transferase mu 1) and aldo-keto-reductase 1C (AKR1C) antisera were kindly provided by Professor John Hayes (University of Dundee, Scotland). Anti-NQO1, anti-AKR1B8, and anti-HO-1 were generated in our laboratory as described previously [39, 40]. DSS (36–50 kD) was from MP Biomedicals (Aurora, OH, USA). Compound C33 was synthesized and purified to >99% purity in this laboratory as described previously [38]. The structures of C33 and RSV are shown in Figure 1(a).

### 2.2. Cell Cultures

Human colon cancer LS174T and Caco2 cells were obtained from the cell bank of the Shanghai Institute of Cell Biology, Chinese Academy of Sciences (Shanghai, China), which also carried out the authentication. The cells were maintained in a growth medium containing Dulbecco's modified Eagle's medium with Glutamax supplemented with 10% fetal bovine serum and antibiotics as described elsewhere [41]. After immediate expanding, multiple aliquots were cryopreserved and cells were used within 6 months after thawing, not exceeding 10 passages. All medium supplements for cell culture were from Invitrogen (Shanghai, China). Cells treated with dimethylsulfoxide (DMSO, 0.1% *v*/*v*) served as negative controls.

### 2.3. Animals

C57BL/6 WT mice were purchased from Shanghai Laboratory Animal Center (Chinese Academy of Sciences, Shanghai, China). *Nrf2^−/−^* mice were kindly provided by Prof. Masayuki Yamamoto (University of Tsukuba, Japan) [2]. All animal procedures were performed with the approval of the Laboratory Animals Ethics Committee of Zhejiang University.

**Figure 2:**
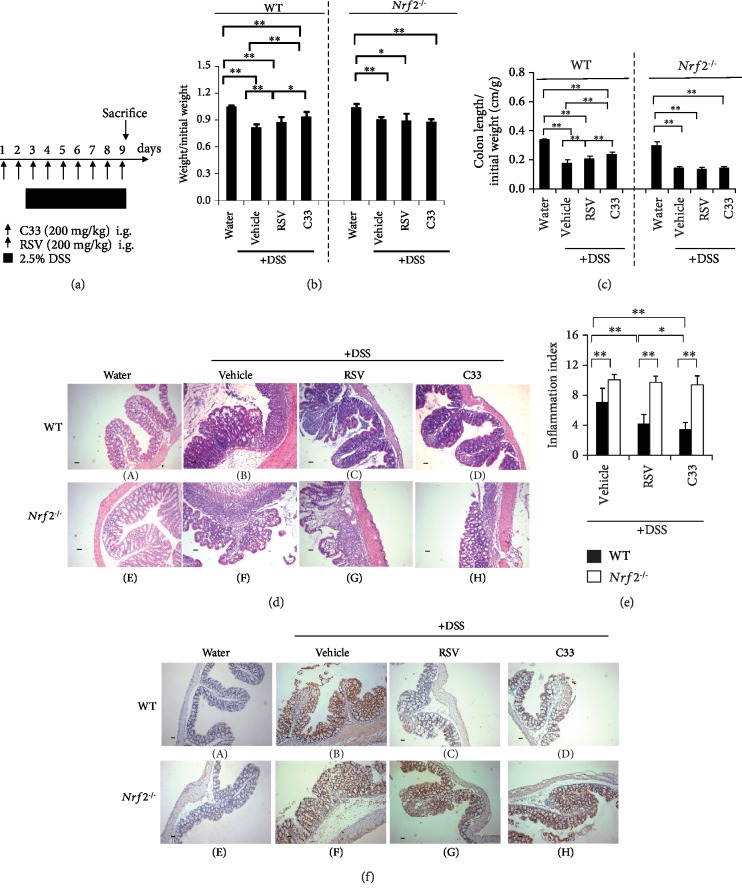
C33 ameliorates DSS-induced colitis in WT but not *Nrf2^−/−^* mice. (a) Experimental protocol for C33 and RSV treatment in DSS-induced colitis. WT and *Nrf2^−/−^* mice were given C33 (200 mg/kg i.g.), RSV (200 mg/kg i.g.), or vehicle (10% ethanol) daily. Two days later, while the mice continued daily treatment with RSV or C33, they were also given normal drinking water or water containing 2.5% DSS for 7 days. (b) Evaluation of the body weight of WT and *Nrf2^−/−^* mice after treatment as in (a). Initial body weight was set as 1. (c) Colon length from mice treated as in (a). Colon length is represented as a ratio (cm/g) relative to the starting weight of mice prior to DSS administration (*n* = 7–9). (d) Representative H&E staining of colon sections from mice treated as in (a) (scale bar, 50 *μ*m; original magnification, ×40). WT, A–D; *Nrf2^−/−^*, E–H. Water (A and E), mice on normal drinking water without any treatment. +DSS (B–D and F–H), mice on drinking water containing 2.5% DSS. (e) Combined scores for the severity of crypt damage (mean ± SD, *n* = 3; ^∗^*p* < 0.05, ^∗∗^*p* < 0.01). (f) IHC staining of colonic sections with anti-nitrogen tyrosine (scale bar, 100 *μ*m; original magnification, ×200). WT, A–D; *Nrf2^−/−^*, E–H. Water (A and E), mice on normal drinking water without any treatment. +DSS (B–D and F–H), mice on drinking water containing 2.5% DSS. The WT mice on normal drinking water (control) was set at 1. Values are mean ± SD (*n* = 5; ^∗^*p* < 0.05, ^∗∗^*p* < 0.01).

**Figure 3:**
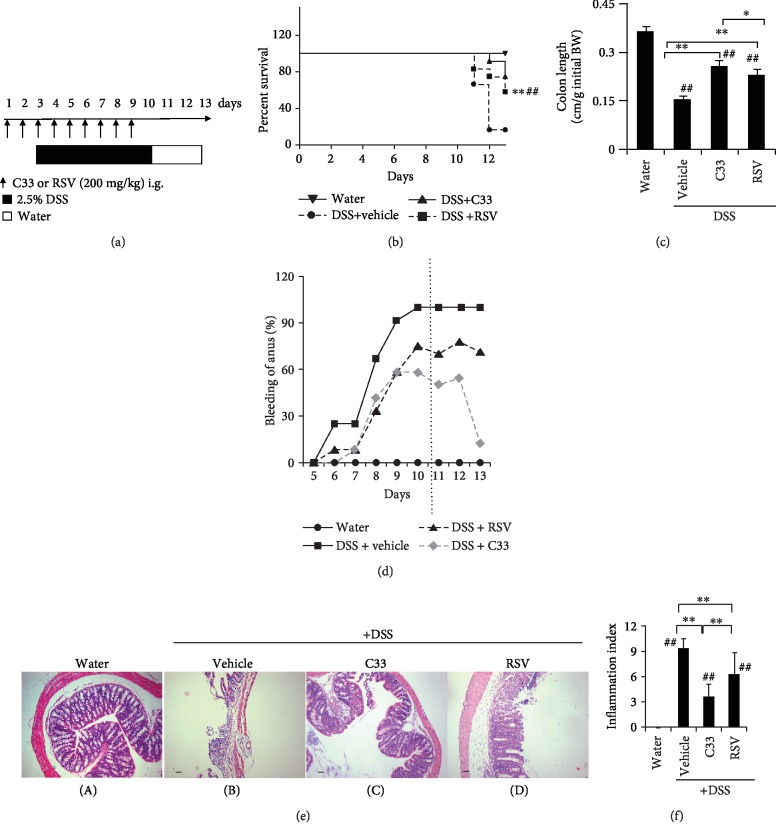
C33 promotes recovery from DSS-induced colitis in WT mice. (a) Experimental protocol for C33 and RSV treatment in DSS-induced colitis. WT and *Nrf2^−/−^* mice were treated with C33 (200 mg/kg i.g.), RSV (200 mg/kg i.g.), or vehicle (10% ethanol) daily. Two days later, while the mice continued with the daily RSV or C33, they were also given normal drinking water or water containing 2.5% DSS for 7 days. After cessation of DSS exposure and RSV or C33 treatment, mice were given water for further three days. (b) Survival rates of the mice. ^∗∗^*p* < 0.01 vs. DSS+vehicle group; ^##^*p* < 0.01 vs. DSS+RSV group. (c) Colon length at the end of experiments represented as a ratio (cm/g) relative to the weight of mice at the start of the experiment (*n* = 3 (water); 3 (vehicle); 9 (C33); 7 (RSV)). (d) The percentage of the mice with anal bleeding during the experiment. (e) Representative images of H&E staining of colon sections from mice in (a) (scale bar, 50 *μ*m; original magnification, ×40). Water (A), mice on normal drinking water without any treatment. +DSS (B–D), mice on drinking water containing 2.5% DSS. (f) Inflammation index of the mice treated as in (a). Values are mean ± SD (*n* = 3–9). ^∗^*p* < 0.05, ^∗∗^*p* < 0.01, difference between the colitis WT mice with different treatments. ^##^*p* < 0.01, difference between the WT mice on normal drinking water without any treatment and the colitis WT mice with different treatments.

In the 3-day duration C33 or RSV experiments, WT and *Nrf2^−/−^* mice were given C33 (200 mg/kg i.g.), RSV (200 mg/kg i.g.), or 10% ethanol (vehicle) daily for 3 days. To induce colitis in mice, male C57BL/6 background WT and *Nrf2^−/−^* mice between 6 and 8 weeks of age were given 2.5% DSS in the drinking water *ad libitum* for 7 days. Mice given regular drinking water throughout the treatment period were used as controls. Previous studies have shown that RSV doses in the range of 15 to 750 mg/kg in the mouse are effective in delaying or preventing carcinogenesis without any toxicity [34]. A dose of 200 mg/kg RSV or C33 was chosen for this study. The protocol for the C33 or RSV treatment experiments with colitis mice is summarized in Figure 2(a). Briefly, male C57BL/6 background WT or *Nrf2^−/−^* mice (6-8 weeks old) were given C33 (200 mg/kg in 10% ethanol, i.g.), RSV (200 mg/kg in 10% ethanol, i.g.), or vehicle (10% ethanol) daily, starting 2 days before the administration of DSS (2.5%) in the drinking water for 1 week, until the termination of the experiment after 7 days of DSS administration (*n* = 3–9). In the dose effect experiments, WT mice were treated with C33 or RSV (10–400 mg/kg i.g.) or vehicle (10% ethanol) daily. Two days later, while the mice continued with the daily C33 treatment, they were also given normal drinking water or water containing 2.5% DSS for 7 days. The protocol to assess the effects of C33 or RSV on survival of colitis mice is summarized in Figure 3(a) (*n* = 3–9). As described above, male C57BL/6 background WT or *Nrf2^−/−^* mice (6-8 weeks old) were given C33 (200 mg/kg in 10% ethanol, i.g.), RSV (200 mg/kg in 10% ethanol, i.g.), or vehicle (10% ethanol) daily, starting 2 days before the administration of DSS (2.5%) in the drinking water for 1 week, until the termination of the experiment after 7 days of DSS administration. After cessation of DSS exposure and RSV or C33 treatment, mice were given water *ad libitum* for a further three days. The number of deaths was recorded. At the end of the experiments, mice were sacrificed. At autopsy, the small intestine and large bowel were flushed with saline and excised. After measuring the length of the large bowel, it was cut longitudinally and fixed in 10% formalin before paraffin embedding as previously described [14, 42]. The small intestine and part of the colon were also removed and snap-frozen immediately in liquid N_2_, being stored at -70°C before the preparation of soluble extracts as described previously [43].

For paraffin-embedded tissue, sections cut at 3 *μ*m were stained with hematoxylin and eosin (H&E). The histological score was the sum of scores of four individual inflammatory parameters: inflammation severity (0, 1, 2, or 3), ulceration (0 or 1), inflammation area involved (0, 1, 2, 3, or 4), and hyperplasia and dysplasia (0, 1, 2, or 3) as detailed previously [44].

### 2.4. ELISA

Serum interleukin 6 (IL-6) and tumor necrosis factor alpha (TNF-*α*) concentrations were measured using an RSG ELISA kit (Affymetrix, USA), following the manufacturer's instructions.

### 2.5. Western Blot Analysis

Whole-cell extracts were prepared as described previously [45]. Small intestine and colon extracts were prepared as previously reported [43]. Protein samples were separated on SDS-PAGE gels, and immunoblotting was carried out using the standard protocol. Immunoblotting with antibody against actin was performed to confirm equal loading for whole-cell and tissue extracts. The relative protein levels were calculated by quantification of band intensity with an Odyssey infrared imaging system (LI-COR® Biosciences) and normalized to actin.

### 2.6. Determination of Reduced Glutathione (GSH)

GSH in the small intestine and colon was measured as described previously [46].

### 2.7. Immunohistochemistry (IHC)

IHC of colon sections from mice was carried out using formalin-fixed paraffin-embedded tissue as described previously [47]. The semiquantitative results of IHC were based on the average value from three mice per group. Three separate slides from each mouse were analyzed. Images were captured under a light microscope (Olympus BX41, Shanghai, China). Image Pro Plus 6.0 software (Media Cybernetics, Inc., Rockville, MD, USA) was used to analyze the staining intensity. Five microscopic fields at ×100 magnification were randomly selected, and the integral optical density (IOD) of the protein of interest was calculated, and this was considered to be the expression level.

### 2.8. Real-Time Quantitative PCR (RT-qPCR)

Total RNA isolation and RT-qPCR were performed as described previously [45]. Each assay was performed in triplicate. The results were analyzed with 480II Real-Time PCR System software (Roche). The level of 18S RNA was used as an internal standard.

### 2.9. Statistical Analysis

All statistical analysis was performed using Stata 7 statistical software (StataCorp LLC, College Station, TX, USA). Student's *t*-test for unpaired results was used to evaluate differences between two groups. One-way ANOVA with post hoc Dunnett's test was used to test dose response effect by comparing groups to the control. Log-rank (Mantel-Cox) test was used to evaluate the differences of survival distributions between two groups. *p* < 0.05 was considered to be statistically significant.

## 3. Results

### 3.1. C33 Activates Endogenous ARE-Driven Genes *via* Nrf2

To confirm whether C33 affects the expression of endogenous genes downstream of Nrf2, LS174T cells were exposed to C33 (1 *μ*M), RSV (5 *μ*M), or 20 *μ*M *tert*-butyl hydroquinone (tBHQ, a known Nrf2 activator) for 24 h. Western immunoblotting showed that, like tBHQ, C33 increased the expression of Nrf2 (Figure 1(b), lanes 3 and 4) and mRNA and proteins levels of its target genes AKR1C and NQO1 (Figures 1(c) and 1(d)). Semiquantitative analysis revealed that, at a lower dose of 1 *μ*M, C33 had a stronger effect on the expression of AKR1C and NQO1 than RSV at a higher dose of 5 *μ*M (Figure 1(c), lanes 2 and 3). Similar increased expression of Nrf2, AKR1C, and NQO1 by C33 was also observed in Caco2 cells (SFig. [Supplementary-material supplementary-material-1]). Moreover, we assessed the expression of ARE-driven genes in WT and *Nrf2^−/−^* MEFs treated with C33 (1 *μ*M). Western immunoblotting revealed marked induction of Ho-1 and Nqo1 in response to C33 in WT MEFs (SFig. [Supplementary-material supplementary-material-1], lane 3). In contrast, no such induction occurred in *Nrf2^−/−^* MEFs, indicating that C33 activates ARE-driven genes through Nrf2 *in vitro* (SFig. [Supplementary-material supplementary-material-1], lanes 3 and 7). Again, in WT MEFs, the efficacy of C33 in activating ARE-driven genes was markedly higher than that of RSV (SFig. [Supplementary-material supplementary-material-1], lanes 2 and 3).

### 3.2. C33 Activates the ARE Gene Battery *In Vivo*

To test whether C33 is able to activate the Nrf2/ARE signaling pathway *in vivo*, WT and *Nrf2^−/−^* mice were given C33 (200 mg/kg i.g.) or RSV (200 mg/kg i.g.) for 3 days. Although both compounds significantly increased the protein levels of Gstm1 and Nqo1 in the small intestines from WT mice, but not *Nrf2^−/−^* mice (Figure 1(e)), C33 triggered a stronger induction of the ARE gene products than RSV. At the same dose, Gstm1 was induced 3-fold and Nqo1 8-fold in the small intestine following C33 treatment, but only 1.4- and 5-fold with RSV. Similar stronger induction of Gstm1 by C33 also occurred in the colons from WT mice (Figure 1(f), lane 3). Furthermore, similar to RSV, C33 markedly enhanced the GSH levels in the small intestines and the colons from WT mice (Figure 1(g)). Thus, C33 activates ARE-driven genes *in vivo* through Nrf2.

### 3.3. C33 Alleviates DSS-Induced Colitis in WT Mice

**Figure 4:**
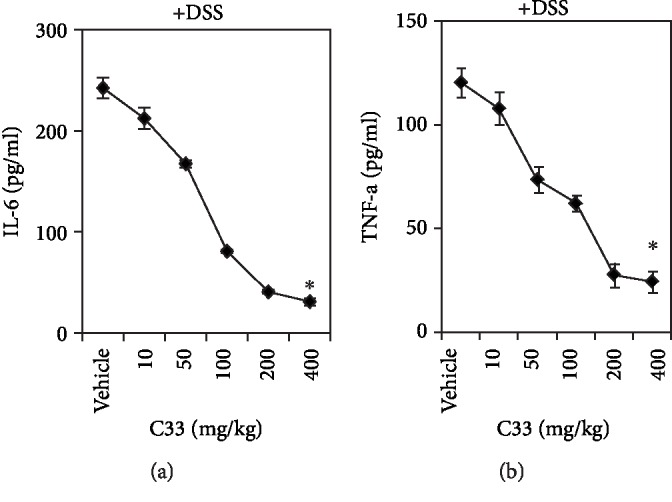
C33 dose-dependently inhibits cytokine production in DSS-induced colitis mice. WT mice were treated with C33 (10–400 mg/kg i.g.) or vehicle (10% ethanol) daily. Two days later, while the mice continued with the daily C33 treatment, they were also given normal drinking water or water containing 2.5% DSS for 7 days. At the end of experiments, blood was taken. Levels of IL-6 and TNF-*α* in plasma were measured by ELISA. Values are mean ± SD (*n* = 4). ^∗^*p* < 0.05, the asterisks indicate a significant difference compared to the vehicle group.

It is well established that RSV has anticolitis properties [32]. To test whether C33 has similar effects, WT and *Nrf2^−/−^* mice were given 2.5% DSS in drinking water for 7 days to induce colitis (Figure 2(a)). The colitis mice of both genotypes exhibited a dramatic drop in body weight (Figure 2(b)) and a marked colon shortening (Figure 2(c)). Histopathological analysis of the colon sections revealed that the *Nrf2^−/−^* mice displayed more severe features of colitis than the WT mice with regard to epithelial necrosis and the distortion of crypts in ulcerative areas (Figure 2(d)). We found that, similar to RSV, oral supplementation with C33 prior to the DSS treatment attenuated the reduction of body weight and colon length in WT mice (Figures 2(b) and 2(c)). Moreover, the body weight and colon length of WT mice with C33 were significantly greater than those with RSV (Figures 2(b) and 2(c)). Furthermore, like RSV, C33 alleviated the microscopic colon damage as shown by a significant decrease in the inflammation index (Figure 2(e)) and inhibited the expression of nitrotyrosine, which is an indicator of inflammation and forms in the presence of the active metabolite nitric oxide in WT mice (Figures 2(f) C–D). Interestingly, C33 appeared to better protect the colonic mucosal structure, limiting multifocal inflammation to the basal one-third of the mucosa with lost crypts. In contrast, supplementation with C33 and RSV failed to affect the body weight, colon length, and inflammatory features of *Nrf2^−/−^* colitis mice, indicating that Nrf2 is essential for the actions of C33 and RSV against colitis. Furthermore, C33 dose-dependently inhibited the production of IL-6 and TNF-*α* (Figure 4). Taken together, our results demonstrate that C33 has a better anticolitis effect than RSV.

### 3.4. C33 Promotes ARE-Driven Gene Expression and Inhibits NLRP3 Expression in WT Colitis Mice

**Figure 5:**
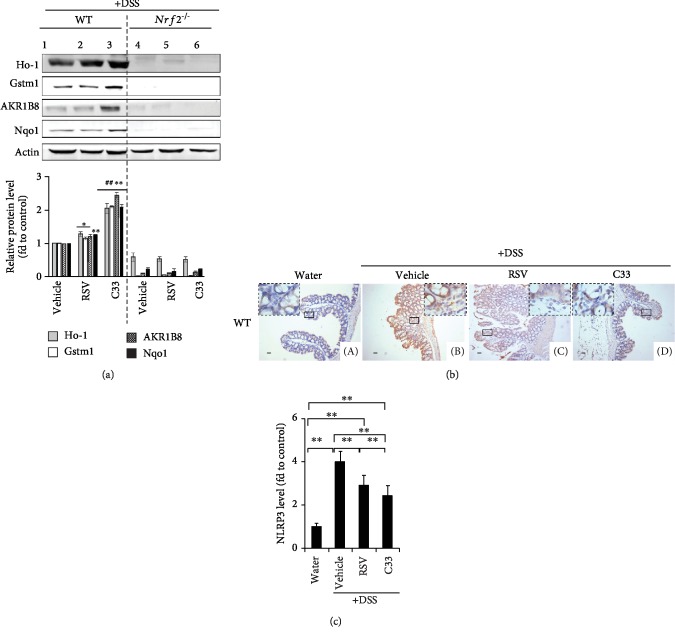
C33 inhibits NLRP3 expression and induces the expression of ARE-driven genes in WT colitis mice. (a) WT and *Nrf2^−/−^* mice were given C33 (200 mg/kg i.g.), RSV (200 mg/kg i.g.), or vehicle (10% ethanol) daily. Two days later, while the mice continued daily treatment with RSV or C33, they were also given normal drinking water or water containing 2.5% DSS for 7 days as in Figure 2(a). Soluble extracts from the colon of mice were analyzed by Western immunoblotting with antibodies against Ho-1, Gstm1, AKR1B8, or Nqo1. Upper panel, representative images of Western immunoblots. Each lane shows the results for a sample from a single mouse. Actin was used as a loading control. Lower panel, semiquantitative result of blot. The value for the same protein from WT mice treated with vehicle (control) was set at 1. Values are mean ± SD (*n* = 3). ^∗^*p* < 0.05, ^∗∗^*p* < 0.01, compared with vehicle-treated mice with same protein and phenotype. ^##^*p* < 0.01, compared with RSV-treated mice with same protein and phenotype. (b) Immunohistochemical analysis of the expression of NLRP3 in the colons from WT mice treated as in Figure 2(a) (scale bars, 100 *μ*m; original magnification, ×200). A: mice on normal drinking water without any treatment. B: mice treated with vehicle and on drinking water containing 2.5% DSS. C: mice treated with RSV and on drinking water containing 2.5% DSS. D: mice treated with C33 and on drinking water containing 2.5% DSS. Images represent results from three separate experiments. (c) Semiquantitative results from (b). The WT mice on normal drinking water without any treatment (control) was set at 1. Values are mean ± SD, *n* = 3; ^∗∗^*p* < 0.01.

To gain a better understanding of the anticolitis effect of C33, soluble protein extracts were prepared from the colons of WT and *Nrf2^−/−^* colitis mice. Western immunoblotting revealed that, while both RSV and C33 maintained high expression levels of Ho-1 in WT colitis mice, C33 induced higher expression of Gstm1, AKR1B8, and Nqo1 proteins than RSV (Figure 5(a), lanes 2 and 3). NLRP3 plays a critical role in the inflammatory response and is regulated by Nrf2 [48, 49]. In the colons from control mice, the NLRP3 staining was weak in the goblet cells (Figure 5(b) A). In DSS-induced colitis, the expression of NLRP3 was dramatically higher in the epithelial and stromal cells (Figure 5(b) B). However, both RSV and C33 suppressed the NLRP3 expression induced by colitis (Figure 5(b) C and D). Again, C33 showed significantly stronger inhibition of NLRP3 expression than RSV (Figures 5(b) and 5(c)). Our data indicate that C33 is able to inhibit induction of the inflammasome.

### 3.5. C33 Improves Survival of Colitis Mice

**Figure 6:**
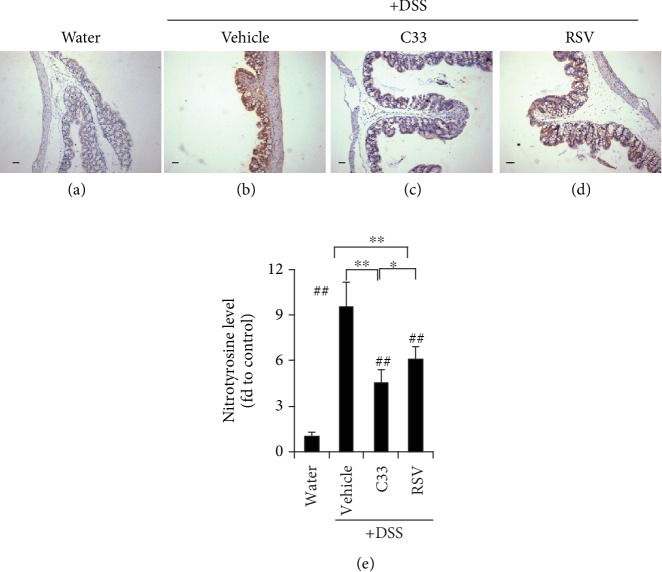
C33 inhibits the expression of nitrotyrosine in colitis mice. IHC staining of nitrotyrosine in colon sections from mice in Figure 3(a). WT mice were treated with C33 (200 mg/kg i.g.), RSV (200 mg/kg i.g.), or vehicle (10% ethanol) daily. Two days later, while the mice continued with the daily RSV or C33, they were also given normal drinking water or water containing 2.5% DSS for 7 days. After cessation of DSS exposure and RSV or C33 treatment, mice were given water for further three days. Water (a), mice on normal drinking water without any treatment. +DSS (b–d), mice on drinking water containing 2.5% DSS. Scale bars, 100 *μ*m; original magnification ×200. (e) Semiquantitative results. The WT mice on drinking water without any treatment (control) was set at 1. Values are mean ± SD (*n* = 3–9). ^∗^*p* < 0.05, ^∗∗^*p* < 0.01, difference between the colitis WT mice with different treatments. ^##^*p* < 0.01, difference between the WT mice on normal drinking water without any treatment and the colitis WT mice with different treatments.

To assess whether C33 has any effect on the survival of colitis mice, the mice received normal drinking water for a 3-day recovery period after the 7 days of DSS colitis induction (Figure 3(a)). While the vehicle group showed a survival rate of 25%, the survival rate for the RSV group was 58.3% and for C33 75% (Figure 3(b)). Although both RSV and C33 prevented shortening of the colon, the colon was significantly longer in the C33-treated mice than in the RSV-treated mice (Figure 3(c)). While anal bleeding in the vehicle group reached 100% during the 3 days after the DSS administration was stopped, only 70-78% showed this in the RSV group. Strikingly, in the C33 group, anal bleeding was only 50-55% on day 11 and day 12 and only 13% on day 13 (Figure 3(d)). Histological examination of colonic tissues revealed that the colonic epithelium in the vehicle group suffered more severe damage with a loss of crypt architecture, and extensive crypt injury and inflammation (Figure 3(e) B), than the C33 and RSV groups (Figure 3(e) C and D). While both C33 and RSV markedly reduced the colitis score, the C33 group had a significantly lower score than the RSV group (Figure 3(f)). Although both C33 and RSV markedly reduced the expression level of nitrotyrosine in the WT mice, significantly weaker staining for nitrotyrosine was observed in the colons from C33-treated colitis mice (Figure 6(c)). Taken together, our data indicate that C33 has a better effect on the recovery of colitis than RSV.

## 4. Discussion

In a previous study, we developed and identified C33 as a novel activator of Nrf2. Here, we carried out further studies to assess the anti-inflammatory potential of the compound. We demonstrated that, without any biochemical toxicity, C33 targeted the Nrf2 signaling pathway specifically *in vivo* and was effective in the amelioration of colitis.

We found that C33 upregulated a wide range of endogenous Nrf2 target genes *in vitro* and *in vivo* with greater potency than its parent compound RSV. Upregulation of the ARE gene battery by C33 was abolished in *Nrf2^−/−^* mice, indicating that C33 targets the Nrf2 signaling pathway *in vivo*.

Here, we used the mouse model of colitis induced by DSS to study the anticolitis properties of C33. A loss of body weight and shortened colon are characteristic features of this model. We found that treatment with RSV or C33 attenuated the weight loss and colon shortening in WT mice. The C33 group showed better effects than the RSV group in maintaining body weight and colon length. Furthermore, as expected, RSV markedly reduced the inflammation index. Interestingly, this index in the C33 group was significantly lower than that in the RSV group. Moreover, while the levels of nitrotyrosine were significantly decreased in the RSV and C33 groups, their levels in the C33 group were lower than those in the RSV group. Moreover, C33 dose-dependently inhibited the production of cytokines IL-6, TNF*α*, and the NLRP3 inflammasome. Importantly, the anticolitis effects of C33 and RSV were abolished in the *Nrf2^−/−^* mice, indicating that activation of the Nrf2/ARE signaling pathway is critical for their anticolitis effects.

In this study, we also investigated the effect of C33 on the survival rate of colitis mice. After 3 days of recovery after stopping the DSS administration, the C33 treatment group had less severe microscopic injury in the colon, indicating better recovery from inflammation, than the RSV group. In addition, C33 treatment prevented weight loss. RSV and C33 treatment increased the survival rate to 58% and 75%, respectively. Taken together, these findings suggested that the improved survival rate of C33-treated mice is possibly due to the fact that C33 is more effective in suppressing or limiting the development of DSS-induced colitis, histological inflammation, and shortening of the colon.

Various members of the NLR family contribute to the production and secretion of cytokines [50]. Notably, NLRP3 inflammasome has been shown to be closely associated with Crohn's disease [51]. Bauer et al. reported that DSS causes lysosomal damage and activates NLRP3 inflammasomes [52]. Recently, Liu et al. found that activation of Keap1-Nrf2-ARE signaling inhibits NLRP3, leading to the alleviation of DSS-induced acute colitis [49]. 3-(2-Oxo-2-phenylethylidene)-2,3,6,7-tetrahydro-1H-pyrazino[2,1-a] isoquinolin-4(11bH)-one, a novel potent Nrf2/ARE inducer, has been found to protect against DSS-induced colitis *via* inhibiting the NLRP3 inflammasome [53]. Our results clearly demonstrated the ability of C33 to inhibit the NLRP3 inflammasome.

The trihydroxystilbene scaffold of RSV has been the subject of synthetic manipulation by medicinal chemists with the aim of improving the pharmacokinetic properties of the compound. These attempts at chemical synthesis have predominantly been concerned with the introduction of additional hydroxy moieties into the trihydroxystilbene framework and with various degrees of phenol group methylation. Structure activity studies have shown that the introduction of methoxy groups in place of hydroxy moieties increases the stability of the molecule, making it less susceptible to phase II conjugation reactions *in vivo*. In addition, methoxy groups added to the stilbene backbone of RSV may enhance its cytotoxicity [54]. It has also been revealed that methoxy moieties at positions 3,5- and 3,4,5- of the trihydroxy stilbene framework play a crucial role in the proapoptotic activity of the molecule [55]. A methylated analogue of RSV, 3,4,4′,5-tetramethoxystilbene (DMU-212) has been shown to have antiproliferative effects in cancer cell lines and displays antitumor activity in animal models of cancer [56–60]. To our knowledge, this is the first study to evaluate the properties of an imine analog of RSV on Nrf2 signaling and anticolitis activity. While the work described here not only defines molecular alterations that improve the potency of the RSV molecule in activating the Nrf2 signaling pathway, it suggests structural features that can be altered without abrogating its anticolitis ability in a mouse model. Such knowledge may help guide synthetic strategies to improve the pharmacologic properties of RSV.

## Data Availability

No data were used to support this study.

## Abbreviations

AKR: Aldo-keto reductase
ARE: Antioxidant response element
DSS: Dextran sulfate sodium salt
GSH: Reduced glutathione
Gstm1: Glutathione S-transferase mu 1
H&E: Hematoxylin and eosin
HO-1: Heme oxygenase-1
IHC: Immunohistochemistry
IL: Interleukin
IRA: Imine resveratrol analog
Keap1: Kelch-like ECH-associated protein 1
NLRP3: NOD-like receptors family pyrin domain containing 3
NQO1: NAD(P)H:quinone oxidoreductase 1
Nrf2: Nuclear factor erythroid 2-related factor 2
RSV: Resveratrol
tBHQ: *tert*-Butyl hydroquinone
TNF-*α*: Tumor necrosis factor alpha
WT: Wild-type.

## Supplementary Materials

Supplementary Materials SFig. 1: C33 increases the expression of AKR1C and NQO1 in Caco2 cells more strongly than RSV. Caco2 cells were treated with C33 (1 *μ*M), RSV (5 *μ*M), or tBHQ (20 *μ*M) for 24 h. The cell lysates were analyzed by Western immunoblotting with antibodies against Nrf2 (A), AKR1C, or NQO1 (B). Actin was used as a loading control and tBHQ treatment served as a positive control. Upper panel, representative images of Western immunoblots. Lower panel, semiquantitative result of blot. The value from cells treated with DMSO (control) was set at 1. Values are mean ± SD (*n* = 3). ^∗^*p* < 0.05, ^∗∗^*p* < 0.01, compared with cells treated with DMSO with same protein. ^#^*p* < 0.05, ^##^*p* < 0.01, compared with cells treated with RSV with same protein. SFig. 2: C33 increases the expression of Nqo1 and Ho-1 in WT but not in Nrf2^−/−^ MEFs. Wild-type (WT) and Nrf2^−/−^ mouse embryonic fibroblasts (MEFs) were prepared as described previously (Higgins et al., 2009). WT or Nrf2^−/−^ MEFs were exposed to C33 (1 *μ*M), RSV (5 *μ*M), or tBHQ (20 *μ*M) for 24 h. The cell lysates were analyzed by Western immunoblotting with anti-Nqo1 or anti-Ho-1. Actin was used as a loading control. Lower panel, semiquantitative result of blot. The value for the same protein from WT MEFs treated with 0.1% DMSO (control) was set at 1. Values are mean ± SD (*n* = 3). ^∗^*p* < 0.05, ^∗∗^*p* < 0.01, compared with DMSO-treated MEFs with same protein and phenotype. ^##^*p* < 0.01, compared with RSV-treated MEFs with same protein and phenotype.
