# Supplementary material for: Synthetic Imine Resveratrol Analog 2-Methoxyl-3,6-Dihydroxyl-IRA Ameliorates Colitis by Activating Protective Nrf2 Pathway and Inhibiting NLRP3 Expression
Source: Oxid Med Cell Longev. 2019 Nov 21;2019:7180284. doi: 10.1155/2019/7180284 (PMC6914940; doi:10.1155/2019/7180284)

SFig. 1 C33 increases the expression of AKR1C and NQO1 in Caco2 cells more strongly than RSV. Caco2 cells were treated with C33 (1 μM), RSV (5 μM), or tBHQ (20 μM) for 24 h. The cell lysates were analyzed by Western immunoblotting with antibodies against Nrf2 (**A**), AKR1C or NQO1 (**B**). Actin was used as a loading control and tBHQ treatment served as a positive control. Upper panel, representative images of Western immunoblots. Lower panel, semi-quantitative result of blot. The value from cells treated with DMSO (control) was set at 1. Values are mean ± SD ($n = 3$). *$p < 0.05$, **$p < 0.01$, compared with cells treated with DMSO with same protein. #$p < 0.05$, ##$p < 0.01$, compared with cells treated with RSV with same protein.

SFig. 2 C33 increases the expression of Nqo1 and Ho-1 in WT but not in *Nrf2*$^{-/-}$ MEFs. Wild-type (WT) and *Nrf2*$^{-/-}$ mouse embryonic fibroblasts (MEFs) were prepared as described previously (Higgins *et al.*, 2009). WT or *Nrf2*$^{-/-}$ MEFs were exposed to C33 (1 μM), RSV (5 μM), or tBHQ (20 μM) for 24 h. The cell lysates were analyzed by Western immunoblotting with anti-Nqo1 or anti-Ho-1. Actin was used as a loading control. Lower panel, semi-quantitative result of blot. The value for the same protein from WT MEFs treated with 0.1% DMSO (control) was set at 1. Values are mean ± SD. ($n = 3$). *$p < 0.05$, **$p < 0.01$, compared with DMSO-treated MEFs with same protein and phenotype. ##$p < 0.01$, compared with RSV-treated MEFs with same protein and phenotype.

Higgins, L. G., Kelleher, M. O., Eggleston, I. M., Itoh, K., Yamamoto, M., and Hayes, J. D. (2009). Transcription factor Nrf2 mediates an adaptive response to sulforaphane that protects fibroblasts in vitro against the cytotoxic effects of electrophiles, peroxides and redox-cycling agents. *Toxicol Appl Pharmacol* **237**, 267-280.

SFig. 1

(A)

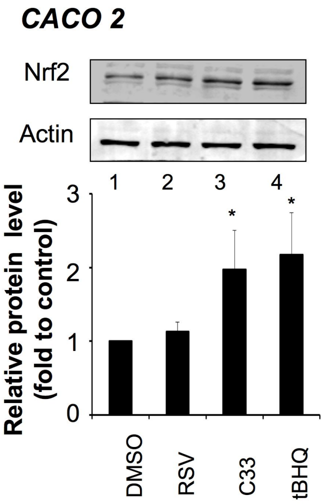

(B)

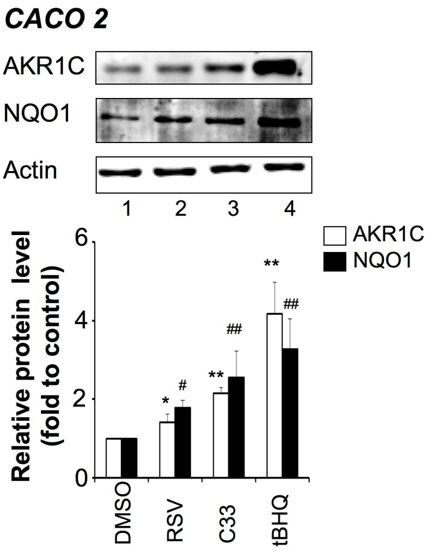

SFig. 2

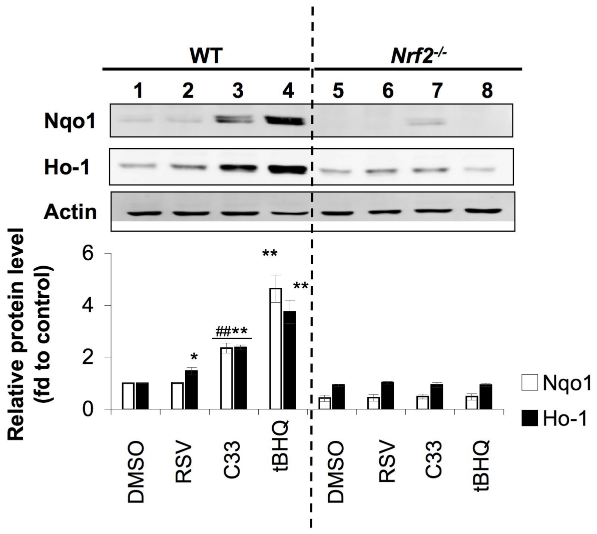

Supplement: Supplementary Materials — SFig. 1: C33 increases the expression of AKR1C and NQO1 in Caco2 cells more strongly than RSV. Caco2 cells were treated with C33 (1 μM), RSV (5 μM), or tBHQ (20 μM) for 24 h. The cell lysates were analyzed by Western immunoblotting with antibodies against Nrf2 (A), AKR1C, or NQO1 (B). Actin was used as a loading control and tBHQ treatment served as a positive control. Upper panel, representative images of Western immunoblots. Lower panel, semiquantitative result of blot. The value from cells treated with DMSO (control) was set at 1. Values are mean ± SD (n = 3). ∗p < 0.05, ∗∗p < 0.01, compared with cells treated with DMSO with same protein. #p < 0.05, ##p < 0.01, compared with cells treated with RSV with same protein. SFig. 2: C33 increases the expression of Nqo1 and Ho-1 in WT but not in Nrf2−/− MEFs. Wild-type (WT) and Nrf2−/− mouse embryonic fibroblasts (MEFs) were prepared as described previously (Higgins et al., 2009). WT or Nrf2−/− MEFs were exposed to C33 (1 μM), RSV (5 μM), or tBHQ (20 μM) for 24 h. The cell lysates were analyzed by Western immunoblotting with anti-Nqo1 or anti-Ho-1. Actin was used as a loading control. Lower panel, semiquantitative result of blot. The value for the same protein from WT MEFs treated with 0.1% DMSO (control) was set at 1. Values are mean ± SD (n = 3). ∗p < 0.05, ∗∗p < 0.01, compared with DMSO-treated MEFs with same protein and phenotype. ##p < 0.01, compared with RSV-treated MEFs with same protein and phenotype. [file 7180284.f1.pdf]
